# Improved vaccination coverage after two rounds of multi-antigenic catch-up vaccination in Mauritania

**DOI:** 10.1371/journal.pgph.0002939

**Published:** 2024-02-14

**Authors:** Maider Pagola-Ugarte, Ankur Rakesh, Julita Gil-Cuesta, David Kidinda, Thierno Moctar Kelly, Sidi Zahaf, Mohamed Mahmoud O. Ely Mahmoud, Mohamedou O. Mohamed Salem, Mbareck Houmeid, Dah Cheikh, Naceredine Ouldzeidoune, Catherine Bachy

**Affiliations:** 1 Luxembourg Operational Research Unit (LuxOR), Médecins Sans Frontières Luxembourg, Luxembourg; 2 Médecins Sans Frontières, Mauritania; 3 Ministry of Health, Mauritania; 4 Department of Expanded Programme of Immunisation, Ministry of Health, Mauritania; 5 AMP Health, Mauritania; 6 World Health Organisation, Mauritania; 7 Médecins Sans Frontières, Operational Centre of Brussels, Belgium; PLOS: Public Library of Science, UNITED STATES

## Abstract

Although Mauritania carried out its Expanded Programme on Immunization (EPI), in 2015 the goal of vaccination coverage (VC) remained unmet in Bassikounou district and Mbera camp, contexts with large migrant populations. In response, during 2018, the national authorities, together with Médecins Sans Frontières organised two rounds of multi-antigenic mass vaccination campaigns (2RMASVC). The campaigns included oral polio (OPV), pneumococcal (PCV13), pentavalent and rotavirus vaccines for all eligible children six weeks to 59 months old. This study describes the results of the 2RMASVC. Cross-sectional household VC surveys (VCS1 and VCS2) were conducted before and after the 2RMASVC. Data were collected on vaccination status according to self-reporting and vaccination cards, and on reasons for non-vaccination (RNV). In total, 4,569 children received at least one dose of vaccine in the first round and 5,602 children in the second. Baseline VC, as fully vaccinated, according to VCS1, was 59.9% of children 12 to 59 months in Bassikounou district and 65.8% in Mbera camp. After the 2RMASVC, the coverages increased to 84.7% and 75.9% respectively. Absence from home, lack of motivation, late initiation of vaccinations and lack of awareness about vaccination were the main RNV during the 2RMASVC. Although the 2RMASVC did not reach its goal of 90%-95% VC, the strategy significantly increased VC in the two settings for children aged 12 to 59 months. Therefore, this catch-up approach could be considered to improve VC of children who miss out of the EPI strategy in resource-limited settings.

## Introduction

Vaccination coverage (VC) of children is an important indicator to assess the protection of a specific population against vaccine preventable diseases and document the performance of national health and immunisation systems [1]. In 2015, the Ministry of Health (MoH) of Mauritania set the goal of immunising 95% of children below 12 months of age at national level, and at least 80% of children at district levels against the diseases targeted by the national Expanded Programme on Immunisation (EPI) [1]. Nevertheless, there were large differences in the EPI coverage estimates among different regions of the country. The Hodh Ech Chargui governorate, bordering Mali, reported one of the lowest vaccination coverages in children 12 to 23 months [2]. This region has seen an influx of 86,000 Malian refugees since 2012; 57,000 of whom had settled in and around Mbera camp [3, 4].

In response, from March 2012 to December 2018, Médecins Sans Frontières (MSF), an international medical humanitarian organisation, provided support to primary and secondary health care in the district of Bassikounou and Mbera camp [5]. In 2018, national authorities and MSF collaborated to conduct two rounds of multi-antigenic mass vaccination campaigns (2RMASVC) in Bassikounou district and Mbera camp, in line with the national Multi-Year Plan for the EPI 2016–2020 [6]. The campaigns were designed to provide two new opportunities for vaccination to children who had missed vaccinations routinely offered by the EPI. Mauritania, like other low-resourced, complex, emergency settings, had limited data sources which could be used to accurately estimate their vaccination coverage [7–9]. Therefore, a baseline vaccination coverage survey (VCS1) was organised to provide reliable information on vaccines needed in the campaigns.

Following the VCS1 findings, two multi-antigenic vaccination campaigns for children aged six weeks to 59 months were carried out. Two months later, a post-campaign vaccination coverage survey (VCS2) was conducted to assess the impact of the campaign.

The 2RMASVC objectives were set at 95% of children to be fully immunised in Bassikounou district and 90% in Mbera camp.

Multi-antigenic catch-up vaccination campaigns in multiple rounds are usually implemented in long-lasting conflict settings, where access to vaccination services has been severely compromised for a long time. However, existing literature on such interventions is limited [10]. This study aims to describe the impact of these campaigns, especially on highly mobile populations and in two types of settings: a rural district and a refugee camp. The presence of baseline and post-intervention VC estimates in the district of Bassikounou and in the refugee camp of Mbera were a golden opportunity to objectively document the added value of this type of vaccination strategy in both settings.

With the most recent immunisation data from 2011–2015, this study provided an update on the vaccination status of children in Bassikounou district [6, 11]. Furthermore, to the best of our knowledge, the vaccination coverage of oral polio, pentavalent, pneumococcal and rotavirus vaccines amongst the population in Mbera camp had never been reported.

The outcomes of this study could help national authorities and other humanitarian agencies consider implementing similar vaccination strategies.

## Methods

### Study design

This was a descriptive study of two cross-sectional household vaccination coverage surveys with random two-stage cluster sampling. The surveys took place in the Bassikounou district and in the Mbera camp preceding and following the multi-antigenic mass vaccination campaigns.

### Vaccination campaigns and survey procedures

The 2RMASVC followed the national EPI schedule for children six weeks to 11 months [6] and the WHO vaccination catch-up schedule for children 12 to 59 months [12] as validated by the Mauritanian health authorities [6]. Measles vaccine was not included in the campaigns, as the MoH held a country-wide vaccination campaign for measles and rubella earlier in the year targeting children nine months to 14 years. Since this study campaign targeted children six weeks to 59 months, vaccines given before 6 weeks of age (BCG, OPV dose 0 and Hep B birth dose) were also excluded.

VC surveys were carried out in February and December 2018, with the 2RMASVC occurring in between that period (round one: 6–17 August; round two: 18–28 September). Training of the health promoters, community leaders and vaccination teams was conducted prior to the start of the campaign, followed by sensitisation activities before each round of multi-antigenic mass vaccination campaigns. As fewer than expected children were vaccinated during the first round of the campaign, the sensitisation activities were heightened, with the messaging adapted to provide more comprehensive information about the campaign to the target families. Vaccines were administered in four fixed sites and by two mobile vaccination teams in Bassikounou, and in five general distribution centres and three schools in Mbera.

### Study population

In both VCS1 and VCS2, all children aged between six weeks and 59 months from Bassikounou and Mbera were eligible to participate. Eligibility of each child for a dose of vaccine was based on the vaccination card or reported vaccination history by the caregiver.

### Data collection

In VCS1, six teams of two members each carried out the interviews in the local languages in Bassikounou and Mbera, and encoded data on a standardized questionnaire in French (S1 and S2 Tables). To prevent conflict of interest, 36 surveyors who did not participate in the 2RMASVC were hired for VCS2. They were allocated to 18 teams of two members each who carried out the survey in Bassikounou and in Mbera.

Guardians were asked how many times they brought the child to the health centre for vaccination and anatomical sites were used to ascertain vaccination history. When the child possessed a card, its content was considered to represent the complete and definitive vaccination history. Results obtained solely by vaccination card are included as supplementary information (S3 Table).

Data on reasons for non-vaccination were collected through an open question to the caregiver. The surveyor then classified the answer according to a list of reasons for non-vaccination that had been previously created (S2 Table). Reasons for non-vaccination were documented for each missed dose of vaccine proposed in the 2RMASVC. The reasons mentioned in the baseline survey are described as supplementary information (S1 and S2 Figs).

### Data entry and analysis

Completeness of the vaccination status was evaluated in children 12 to 59 months who have aged out of the EPI. A child was considered fully vaccinated if she/he had received all eligible vaccines proposed in the 2RMASVC according to the vaccination calendar at the time of vaccination (Table 1). As rotavirus vaccine was introduced later in the national calendar; children >37 months of age at VCS1 and >47 months of age at VCS2 never had a chance to receive this vaccine. Children were defined as partially vaccinated if they were missing at least one dose of the vaccines proposed. Unvaccinated children were those who never received any dose of these vaccines.

**Table 1:** Children aged 12 to 59 months eligible for vaccination during 2RMASVC.

Age group	OPV-1	OPV-2	OPV-3	Penta-1	Penta-2	Penta-3	PCV13-1	PCV13-2	PCV13-3	Rotavirus-1	Rotavirus-2
12–37 months	E	E	E	E	E	E	E	E	E	E	E
38–47 months	E	E	E	E	E	E	E	E	E	E VCS2	E VCS2
48–59 months	E	E	E	E	E	E	E	E	E	NE	NE

Abbreviations: E, Eligible. NE, Not eligible. E VCS2, Eligible during VCS2

For vaccine coverage, results by vaccines were stratified into two age groups (six weeks-11 months and 12–59 months). Reasons for non-vaccination were analyzed for children six week to 59 months.

Data collected on paper were encoded onto an Excel 2010 (Microsoft corporation) database. We used Epi-Info (CDC, Atlanta, USA) software version 7.2.0.1 for the analysis and the 95% symmetric Wald-type confidence intervals. Data quality was ensured through adequate training of the interviewers and data audits during data collection and encoding.

### Sample size

The sample size calculation was carried out with the software "Open Epi" Version 3.01 [13], based on an estimated population of 9,328 children under 59 months in Mbera camp provided by the UNHCR, and a 2016 projection of 9,979 children in Bassikounou by the National Office of Statistics [14, 15]. For VCS1, the sample size used an estimated proportion of fully vaccinated (p) of 60% in Bassikounou and 50% in Mbera, anticipating a design effect (DEFF) of three in Bassikounou and two in Mbera, a desired accuracy (d) of 6% and 5% in Bassikounou and Mbera respectively, and choosing a 5% error (α). Thus, a sample of 750 children was required in Bassikounou and 738 children in Mbera for VCS1.

For the VCS2, carried out two months after the 2RMASVC, ‘p’ was estimated to be 70% for both settings. Choosing an ‘α’ of 5%, a DEFF of two with a ‘d’ of 5%, the minimum sample size required was 632 children in Bassikounou and 616 children in Mbera.

All children aged six weeks to 59 months of each household visited were included. The sample size was divided in 45 clusters of 15 children in Bassikounou and 17 in Mbera for VCS1. For VCS2, 45 clusters of 14 children were needed to complete the sample size in both areas. The cluster selection was repeated in its entirety for the second survey.

### Sampling

A two-stage cluster sampling method was used. In the first stage, the allocation of 45 clusters was performed through systematic sampling with probability of allocation proportional to the population size of all villages in Bassikounou and all blocks in Mbera. In the second stage, the WHO-EPI methodology was adapted to randomly select households within each cluster and locality [16].

If the sample size was not reached after all the households in the cluster were included, the survey team went to the nearest locality or block and sampled until the necessary number of children per cluster were surveyed. In polygamous families or plots with more than one household, one household was randomly chosen. If a visited household was empty, a new household was selected at random as a replacement.

### Ethics

The VC surveys were approved by the Ministry of Health and National Statistical Office of Islamic Republic of Mauritania. The studies fulfilled the exemption criteria set by the MSF Ethical Review Board (ERB) for standardized vaccination coverage surveys. They were conducted with permission from the Medical Director of MSF in Brussels. Authorisation was obtained from the chief of each the village where the survey was carried out. Verbal consent was obtained from each caregiver interviewed. No personal identification was recorded.

## Results

In VCS1, 758 children in Bassikounou and 748 children in Mbera were included; out of those children 37% had vaccination cards in Bassikounou and 42% in Mbera. VCS2 included 646 children in Bassikounou and 641 children in Mbera; 60% possessed vaccination cards in Bassikounou and 66% in Mbera. The population of the two surveys displayed similar characteristics (S4 Table).

During 2RMASVC, a total of 9,437 doses of OPV, 7,324 doses of pentavalent, 7,516 doses of PCV13 and 1,107 doses of rotavirus vaccines were administered to 4,569 children during round one and to 5,602 during round two (S5 Table).

### Vaccination status

By VCS2 the number of unvaccinated children had dropped by 12.3 points in Bassikounou and 9.6 points in Mbera (Table 2).

**Table 2:** Vaccination status in Bassikounou district and Mbera camp for children 12–59 months (unvaccinated, partially or fully vaccinated), Mauritania 2018.

	Vaccination status—Children aged 12 to 59 months									
	Bassikounou					Mbera				
	VCS1		VCS2		P-value	VCS1		VCS2		P-value
	%	95% CI	%	95% CI		%	95% CI	%	95% CI	
Unvaccinated	17.8	12.8–22.8	5.5	2.8–8.4	0.001	16.5	12.2–20.8	6.9	3.9–9.8	0.001
Partially vaccinated	22.4	16.9–27.9	9.8	6.1–13.4	0.001	17.7	13.7–21.7	17.3	13.7–20.8	> 0.05
Fully vaccinated	59.9	52.5–67.3	84.7	80.4–89.0	0.001	65.8	61.1–70.6	75.9	71.1–80.7	0.001
	*Deff*: *2*,*6*		*Deff*: *2*.*1*			*Deff*: *2*.*0*		*Deff*: *1*.*7*		

Abbreviations: VCS1, Vaccination Coverage Survey 1. VCS2, Vaccination Coverage Survey 2. Deff, Design effect.

### Vaccination completeness by vaccine

In both settings, uptake of the last dose of each vaccine had increased in children aged 12 to 59 months by VCS2 (Table 3), but the increases were higher in Bassikounou than in Mbera (27–30% vs 10–15%). However, the baseline VC for children 12 to 59 months was greater in Mbera than in Bassikounou.

**Table 3:** Comparison of VC by vaccine and dose in Bassikounou district and Mbera camp by VCS1 and VCS2, children aged 12 to 59 months, Mauritania 2018.

Children aged 12 to 59 months														
Vaccine	Bassiknou							Mbera						
	VCS1			VCS2			P-value	VCS1			VCS2			P-value
	%	95% IC	DEFF	%	95% IC	DEFF		%	95% IC	DEFF	%	95% IC	DEFF	
**OPV-1**	81.4	76.2–86.6	2.6	94.5	91.6–97.3	2.1	< 0.001	82.5	78.0–87.0	2.0	92.9	89.9–96.0	1.7	< 0.001
**OPV-2**	72.7	66.6–78.7	2.7	90.4	87.1–93.7	1.7	< 0.001	76.9	72.5–81.3	1.6	86.1	81.3–90.8	2.4	< 0.001
**OPV-3**	65.8	59.2–72.5	2.9	84.9	80.6–89.2	1.9	< 0.001	70.7	65.8–81.3	1.6	78.5	73.6–83.4	1.8	< 0.001
**Penta-1**	82.1	77.0–87.1	2.6	94.5	91.6–97.3	2.1	< 0.001	83.2	78.8–87.5	1.9	92.7	89.8–95.7	1.7	< 0.001
**Penta-2**	73.7	67.8–79.6	2.7	88.8	85.4–92.1	1.5	< 0.001	78.1	73.7–82.5	1.7	83.7	78.8–88.6	2.2	< 0.001
**Penta-3**	66.9	60.3–73.5	2.9	84.9	80.6–89.2	1.9	< 0.001	71.0	66.1–75.9	1.7	78.2	73.1–83.2	1.8	< 0.001
**PCV13-1**	81.6	76.4–86.7	2.6	94.5	91.6–97.3	2.3	< 0.001	82.8	78.6–87.0	1.8	92.0	88.9–95.0	1.6	< 0.001
**PCV13-2**	73.0	66.9–79.1	2.7	89.0	85.7–92.2	1.4	< 0.001	77.1	72.8–81.4	1.5	83.9	79.2–88.6	2.0	< 0.001
**PCV13-3**	66.1	59.4–72.7	2.9	84.9	80.6–89.2	1.9	< 0.001	70.7	65.8–75.5	1.6	78.2	73.3–83.0	1.7	< 0.001
**Rotavirus-1**	76.1	69.3–82.9	2.3	91.2	87.7–94.7	1.4	< 0.001	80.8	77.1–84.4	1.0	86.8	82.2–91.4	1.6	< 0.001
**Rotavirus-2**	68.4	61.9–74.9	1.8	88.7	85.1–92.4	1.2	< 0.001	72.3	67.5–77.1	1.2	82.8	77.6–88.0	1.7	< 0.001

Abbreviations: VCS1, Vaccination Coverage Survey 1. VCS2, Vaccination Coverage Survey 2. OPV -1;3, Oral Polio Vaccine dose 1 to 3. Penta-1;3, Diphtheria, Tetanus, Pertussis, Hepatitis B and Haemophilus Influenzae type B vaccine dose 1 to 3. PCV13-1;3, Pneumococcal vaccine dose 1 to 3. Rotavirus- 1;2 rotavirus vaccine dose 1 to 2.

Among children six weeks to 11 months old (S6 Table) there were no increase in coverage for any vaccine dose, but the sample size was too small to detect significant differences (S4 Table).

### Reasons for non-vaccination by VCS2

**Fig 1:**
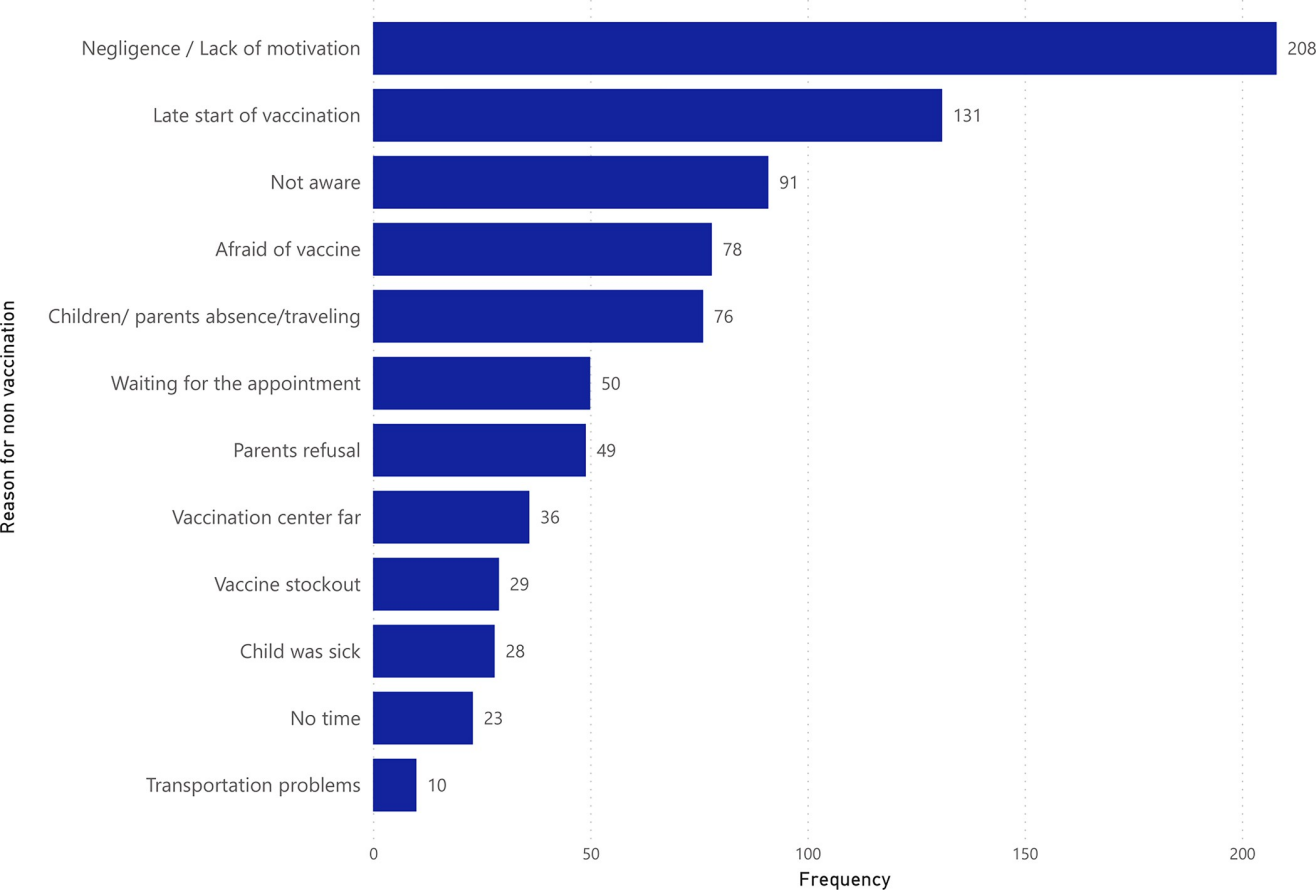
Reasons for non-vaccination for the proposed vaccines among children aged six weeks to 59 months in Bassikounou district reported in VCS2, Mauritania 2018. Each children’s caregiver gave one reason for non-vaccination per dose of vaccine not received. In total 43 children gave 11 reasons for non-vaccination and 99 gave between 1 and 10 reasons for non- vaccination.

**Fig 2:**
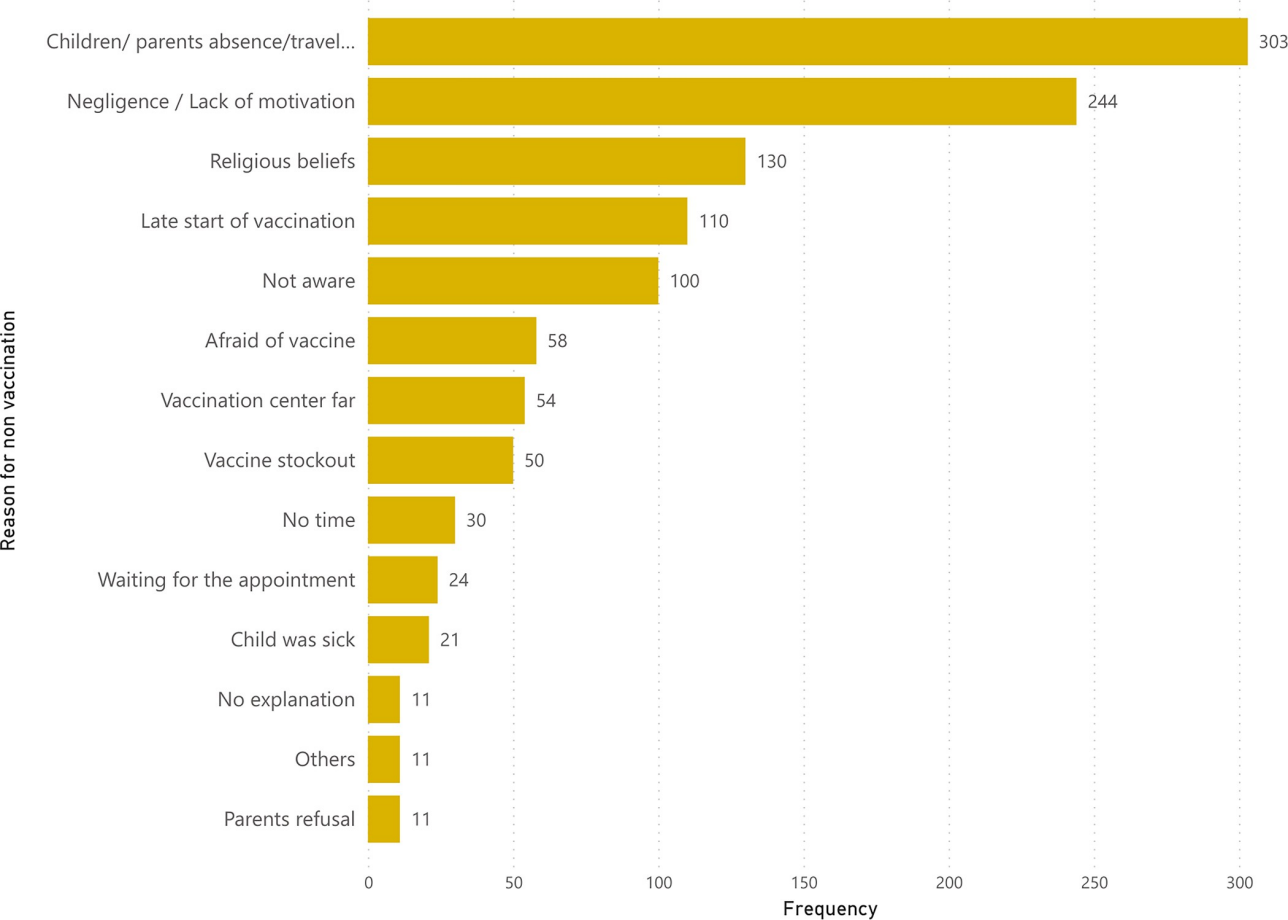
Reasons for non-vaccination for the proposed vaccines among children aged six weeks to 59 months in Mbera camp, reported in VCS2, Mauritania 2018. Each children’s caregiver gave one reason for non-vaccination per dose of vaccine not received. In total 44 children gave between 11 reasons for non-vaccination and 135 gave between 1 and 10 reasons for non- vaccination. Reasons for non-vaccination for seven children were not available for this analysis.

In Bassikounou the most common reasons for non-vaccination were the lack of motivation to get the child vaccinated, followed by the late start of vaccination, caregivers’ lack of awareness about vaccination, fear of vaccines, and children or parents’ absence or travelling (Fig 1). In Mbera the most common reasons were children’s or parent’s absence during the campaign, lack of motivation, religious beliefs, late start of vaccination, and lack of awareness about vaccination (Fig 2).

## Discussion

Our study demonstrates the effectiveness of the multi-antigenic campaign, as the proportion of unvaccinated and partially vaccinated children dropped significantly in both settings. This suggests that such catch-up strategy might be appropriate for the WHO immunisation agenda of 2030, a global strategy to leave no one behind [17] in settings that are unstable or with large numbers of migrants.

Nonetheless, as the proportion of fully-vaccinated children 12 to 59 months only reached 84.7% [CI: 80.4–89.0] in Bassikounou and 75.9% [CI: 71.1–80.7] in Mbera, the campaign did not achieve its goal of 95% and 90% of completely-vaccinated children in these two areas respectively.

This might be partly explained by the fact that only two rounds of the campaign were carried out, and some primary series of vaccines contain three doses. Moreover, among older children, the recommended minimum interval between doses two and three is sometimes longer than the four-week interval between the two vaccination rounds [18]. Furthermore, the sensitisation activities were not optimal in some areas for the first round and had to be intensified before the second round.

Our results showed that baseline VC of children 12 to 59 months was marginally higher in Mbera than in Bassikounou. Similar findings have been reported in other settings, where measles vaccination and nutritional intervention coverages were higher among people living in camps compared to local populations [19, 20]. This could be attributed to the relative ease of conducting mass vaccination campaigns and nutritional interventions in camps rather than in geographically dispersed areas [19]. Large-scale population movements, as observed in districts such as Bassikounou since 2012, further strained the host population’s scarce access to essential services including vaccination [21, 22].

The VC of all scheduled vaccines significantly increased among children aged 12 to 59 months in Bassikounou and in Mbera. The increase was greater in Bassikounou than in Mbera, a difference that could be related to the lower VC at baseline in Bassikounou, leading to a bigger increase with improved access to vaccination activities. Results solely based on vaccination cards showed significantly higher baseline vaccination coverage in Mbera camp. Refugees might be more likely to keep their vaccination cards, as they may be associated with access to other services. These findings were not confirmed by VCS2, where higher VC was seen in Bassikounou than in Mbera (S3 Table).

The high mobility of the population could have influenced the effectiveness of the vaccination campaign, as mobility is associated with lower VC as well as less likelihood to complete vaccination [23, 24]. This was observed in Mbera, where refugees were often absent from the camp during the campaign (Fig 2).

This study failed at showing effectiveness of 2RMASVC in children below 12 months of age, for which other vaccination strategies might be more appropriate. These children are still highly vulnerable to vaccine preventable diseases and vaccinating them is essential to reduce morbidity and mortality [25, 26].

The strength of this study comes from the availability of baseline VC estimates that allowed us to measure the outcome of the intervention. This is rarely possible in humanitarian settings as opportunities to carry out pre-campaign surveys are often limited.

This study is limited by the low proportion of vaccination cards presented during VCS1. This uncertainty around the results is common in low and middle-income countries where the vaccination status of children is based on the recall of the caregivers/parents [27, 28] (S4 Table). This could either lead to an overestimation or underestimation of the true VC due to recall bias [28, 29].

A quarter (26%) of children absent during the campaign helps explain the lower increase in VC in Mbera. Lack of motivation of the parents was one of the main reasons for non-vaccination in both settings, while late start, low awareness of vaccination, religious beliefs and fear of vaccines were reported among the top five reasons despite sensitisation campaigns done by MSF. Poor knowledge of vaccines and vaccination programmes are frequently reported in the literature as key factors that determine vaccination coverage status among children in low and middle-income countries, but they may not be the only factors influencing the choice to vaccinate [23, 24, 30].

Our study shows that multi-antigenic campaigns can improve VC and allow older children to catch up on missed EPI vaccines, although less effectively in highly mobile populations. Multi-antigenic and multiple-round campaigns are more expensive and more difficult to run than those targeting a single disease in one round but allow for a more comprehensive approach. Therefore, adding antigen and a second round to any vaccination campaign should be considered, taking into account the intervention contexts and the beliefs, knowledge and behavior of the communities within.

Naturally, strengthening the activities of the EPI would reduce the need to organise catch-up vaccination campaigns or at least extend the period between these campaigns. Efforts should be made to reduce barriers to routine vaccination including further investment in vaccination sensitisation and increasing accessibility and quality/availability of immunisation services. Integrating vaccination activities with other services such as food distribution would make both interventions more effective. Finally, even though this would probably increase the cost per child vaccinated [10], there is a need to strengthen mobile vaccination strategies to reach remote populations.

The years of experience acquired by MSF in epidemic preparedness allowed us to present this preventive approach of catch-up multi-antigenic vaccination campaigns that since 2014 have been sporadically conducted, but rarely reported [10].

## Conclusion

This study demonstrated that two rounds of multi-antigenic mass vaccination campaigns increased the number of children vaccinated with poliomyelitis, pentavalent, pneumococcal and rotavirus vaccines in a rural community and refugee camp in Mauritania. It halved the number of unvaccinated children in both settings. This preventive approach could be considered as a strategy to improve overall vaccination coverage of children that have been left out by the EPI in resource-limited settings.

## Supporting information

S1 Checklist Inclusivity in global research. (PDF)

S1 Table Vaccination status questionnaire. Standardized questionnaire to obtain vaccination status by vaccine and doses among children aged six weeks to 59 months in Bassikounou district and Mbera camp, Mauritania 2018.
(XLSX)

S2 Table Reasons for non-vaccination questionnaire. Standardized questionnaire to collect the reasons for non-vaccination by vaccine and doses.
(XLSX)

S3 Table Vaccination coverage for vaccination coverage survey 1 (VCS1) and VCS2 in Bassikounou district and Mbera camp among children six weeks to 59 months based on vaccination cards, Mauritania 2018. (XLSX)

S4 Table Descriptive information of the vaccination coverage surveys. Summary table including dates of the surveys, proportion of children which provided the vaccination card (in total and by age group) number of households visited and the number of children included in each survey stratified by age groups in Bassikounou district and Mbera camp, Mauritania 2018.
(XLSX)

S5 Table Number of vaccine doses administered during the two rounds of multi-antigenic mass vaccination campaigns (2RMASVC). Number of Oral polio, Penta, PCV13 and Rotavirus vaccine doses used in the first and second rounds of the 2RMASVC.
(XLSX)

S6 Table Vaccination coverage results for vaccination coverage survey 1(VCS1) and VCS2 in Bassikounou district and Mbera camp among children aged six weeks to 11 months, Mauritania 2018. (XLSX)

S1 Fig Reasons for non-vaccination for the proposed vaccines among children aged six weeks to 59 months in Bassikounou district reported in VCS1, Mauritania 2018. Each children’s caregiver gave one reason for non-vaccination per dose of vaccine not received. In total 115 children gave 11 reasons for non-vaccination and 267 gave between 1 and 10 reasons for non-vaccination.
(TIFF)

S2 Fig Reasons for non-vaccination for the proposed vaccines among children aged six weeks to 59 months in Mbera camp reported in VCS1, Mauritania 2018. Each children’s caregiver gave one reason for non-vaccination per dose of vaccine not received. In total 114 children gave 11 reasons for non-vaccination and 197 gave between 1 and 10 reasons for non-vaccination.
(TIFF)
