# Supplementary figures and images for: Improved vaccination coverage after two rounds of multi-antigenic catch-up vaccination in Mauritania
Source: PLOS Glob Public Health. 2024 Feb 14;4(2):e0002939. doi: 10.1371/journal.pgph.0002939 (PMC10866457; doi:10.1371/journal.pgph.0002939)

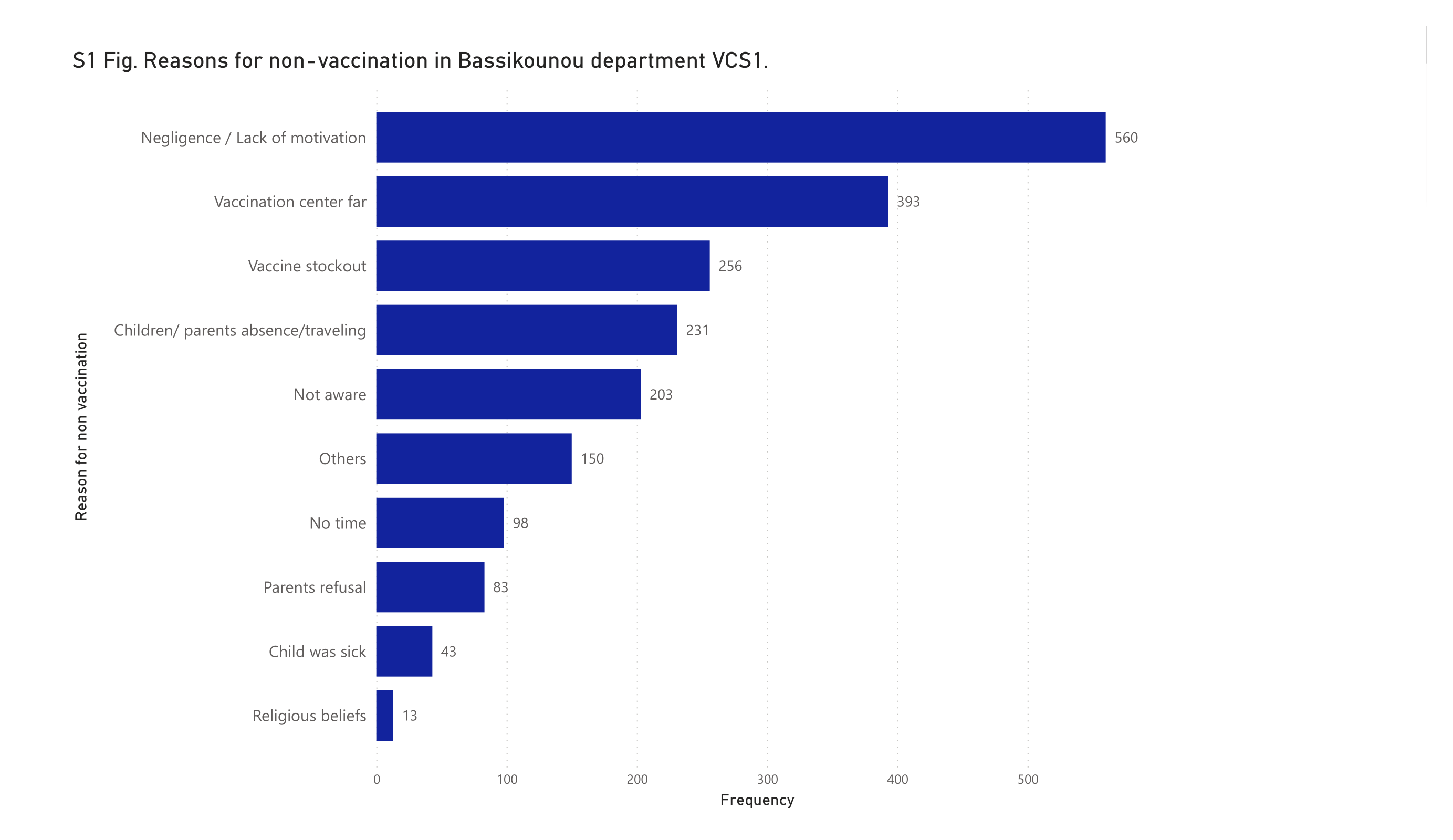

Supplement: S1 Fig — Each children’s caregiver gave one reason for non-vaccination per dose of vaccine not received. In total 115 children gave 11 reasons for non-vaccination and 267 gave between 1 and 10 reasons for non-vaccination. (TIFF) [file pgph.0002939.s008.tiff]

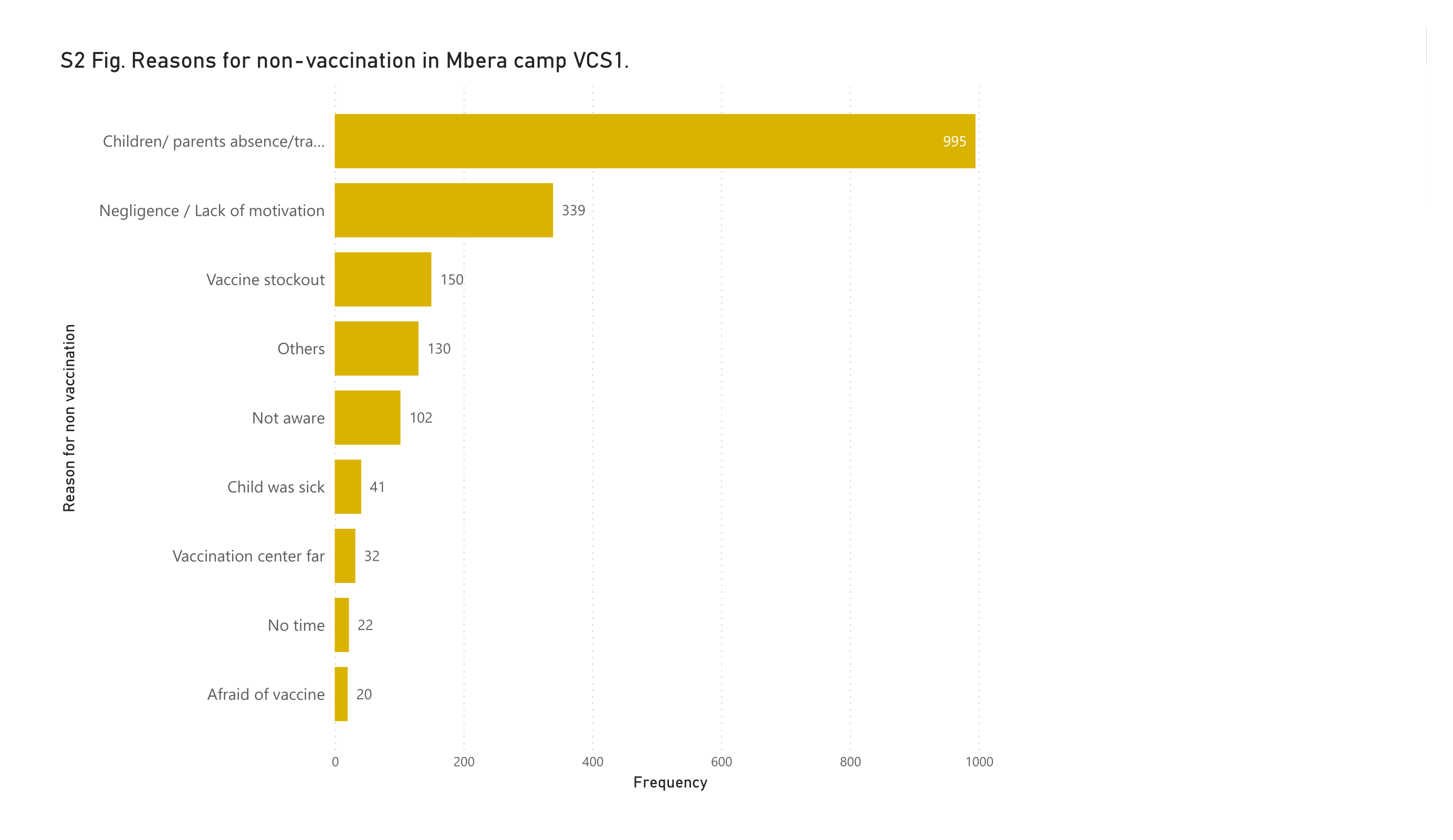

Supplement: S2 Fig — Each children’s caregiver gave one reason for non-vaccination per dose of vaccine not received. In total 114 children gave 11 reasons for non-vaccination and 197 gave between 1 and 10 reasons for non-vaccination. (TIFF) [file pgph.0002939.s009.tiff]
